# Mortality in Two Waves of COVID-19: A Comparative Analysis of a Tertiary Care Hospital in India

**DOI:** 10.7759/cureus.45025

**Published:** 2023-09-11

**Authors:** Saurabh Vig, Jitendra K Meena, Abhishek Kumar, Puneet Rathore, Swati Bhan, Prashant Sirohiya, Gitartha Goswami, Arunmozhimaran Elavarasi, Hari Krishna Raju Sagiraju, Nishkarsh Gupta, Brajesh Ratre, Anuja Pandit, Ram Singh, Balbir Kumar, Rakesh Garg, Ved P Meena, Saurav S Paul, Anant Mohan, Randeep Guleria, Sushma Bhatnagar

**Affiliations:** 1 Onco-Anesthesiology and Palliative Medicine, National Cancer Institute, All India Institute of Medical Sciences (AIIMS), New Delhi, IND; 2 Preventive Oncology, National Cancer Institute, All India Institute of Medical Sciences (AIIMS), New Delhi, IND; 3 Anesthesiology, Sanjay Gandhi Post Graduate Institute of Medical Sciences (SGPGIMS), Lucknow, IND; 4 Onco-Anesthesiology and Palliative Medicine, Dr. B. R. Ambedkar Institute Rotary Cancer Hospital, All India Institute of Medical Sciences (AIIMS), New Delhi, IND; 5 Anesthesiology, Vardhaman Mahavir Medical College (VMMC) and Safdarjung Hospital, New Delhi, IND; 6 Neurology, All India Institute of Medical Sciences (AIIMS) New Delhi, New Delhi, IND; 7 Onco-Anesthesiology and Palliative Medicine, Dr. B. R. Ambedkar Institute Rotary Cancer Hospital, All India Institute of Medical Sciences (AIIMS), Delhi, IND; 8 Anesthesiology and Critical Care, All India Institute of Medical Sciences (AIIMS) New Delhi, New Delhi, IND; 9 Internal Medicine, All India Institute of Medical Sciences (AIIMS) New Delhi, New Delhi, IND; 10 Medicine, All India Institute of Medical Sciences (AIIMS) New Delhi, New Delhi, IND; 11 Pulmonary Medicine, All India Institute of Medical Sciences (AIIMS) New Delhi, New Delhi, IND

**Keywords:** case fatality rate., total mortality rate, covid wave 2, covid wave 1, covid-19

## Abstract

Background

COVID-19 has spread as two distinct surges of cases in many countries. Several countries have reported differences in disease severity and mortality in the two waves.

Objective

Compare the in-hospital mortality in the two COVID-19 waves at a tertiary care hospital in India.

Methods

We conducted a retrospective data collection. Distinct periods of surges in cases and admissions were defined as the first wave spanning from March 2020 to December 2020 and the second wave from April 2021 to June 21, 2021. The primary outcome of this study was to compare mortality rates in terms of total hospital mortality rate (TMR) and case fatality rate (CFR).

Results

Mortality rates of wave 2 were approximately 10 times that of wave 1 (TMR of 20.3% in wave 2 versus 2.4% in wave 1 and CFR of 1.5% versus 17.7% in wave 1 and 2, respectively). Mortalities in wave 2 had a larger proportion of severe disease at presentation, faster progression of symptoms to death, and more patients without any chronic comorbid condition dying due to the direct effect of COVID-19 acute respiratory distress syndrome (ARDS).

Conclusion

Our data matches the worldwide reported pooled hospital mortality figures and shows the comparative difference in disease severity between the two waves.

## Introduction

Countries all over the world are experiencing COVID-19 pandemic surges at different times and with varied severity [1 ]. This disease has spread like wildfire all over the world, affecting more than 220 countries, with more than 204 million reported cases and more than 4 million deaths worldwide [2]. The majority of nations have seen a biphasic spread of this disease with cases peaking in the form of two peaks or waves separated by a trough of low transmission [3-5]. Several countries have reported differences in disease severity, outcomes, and mortality in the two waves of COVID-19 [6].

Like the rest of the world, India experienced an unprecedented surge of cases during the second wave of COVID-19. Despite a significant case burden and limited public health resources, India's case fatality rate (CFR) has been reported to be low, comparable to that of resource-rich countries [7,8]. However, interpreting this data can be challenging. Direct comparisons might be misleading as most estimates are based on population data, which can be influenced by numerous variables such as differences in age distributions between countries and available healthcare resources, among others [9,10]. To the best of our knowledge, no studies from India have directly compared in-hospital mortality across the two waves of COVID-19. Thus, this study aims to describe and compare the in-hospital mortality during the two waves at a tertiary care COVID-19 hospital in India.

## Materials and methods

This study is a retrospective data analysis of hospital records of COVID-19 mortalities. Our hospital, National Cancer Institute, All India Institute of Medical Sciences (AIIMS), New Delhi, catered to COVID-19 cases exclusively from Delhi and adjacent areas. The first COVID-19-positive case was admitted on March 21, 2020, and the first wave of admissions continued till December 2020. There was a transient period of no admissions from January 2021 to March 2021. However, as seen in the rest of the country, a fresh wave of COVID-19 cases was seen in April 2021, and admissions restarted on April 9, 2021. This second wave of admissions continued till July 2021. Thus, we defined the first wave of COVID-19 from March 2020 to December 2020 and the second wave from April to June 2021. Ethical clearance of this study was taken from our institute's ethics committee (ref. no. IEC 07/5.02.2021 RP 35/2021). Inclusion criteria were all mortalities due to COVID-19 in the first and second waves. Retrieved data points were age, sex, symptom details, and oxygen saturation (SpO2) at the time of admission, duration between onset of symptoms and confirmation of COVID-19-positive status, and time between confirmed COVID-19-positive status to hospital admission. Comorbidity status and details on the cause of death were noted, and the total duration of hospital stay was recorded. Exclusion criteria included patients with unknown outcomes or missing primary data points, such as those who were transferred to other hospitals or discharged against medical advice. Cases were categorized into mild-moderate or severe disease based on SpO2 at presentation, following the institutional protocol for classifying COVID-19 severity and its management [11]. The classifications were: mild disease: SpO2 ≥ 94% on room air; moderate disease: SpO2 ranging from 90-93% on room air; severe disease: SpO2 < 90% on room air or patients presenting while on oxygen support. For the final analysis, the cause of death was categorized into two groups. The first was COVID-attributed deaths, where acute respiratory distress syndrome (ARDS) and refractory hypoxia due to COVID-19 were the primary causes of death. The second group comprised deaths attributed to other causes, where COVID-19-related hypoxia was not the primary cause of death but was among the antecedent or contributory factors.

The primary objective of this study was to compare the mortality rates in the form of total hospital mortality rate (TMR) and CFR. Secondary objectives were to compare the demographics, clinical status at admission, comorbidity details, duration of hospital stay, and the main causes of death in the mortalities in the two waves of COVID-19. TMR was defined as the total number of deaths among admitted COVID-19 patients divided by the total number of COVID-19 patients admitted during each wave. CFR was defined as the total number of deaths where COVID-related respiratory failure was the primary cause, divided by the total number of COVID-19 patients admitted during each wave. In both of these calculations, patients whose primary outcome data were unavailable (e.g., those transferred to other hospitals or who left against medical advice (LAMA)) were excluded from the computation of mortality rates. In other words, the denominator used was the total number of admitted patients, excluding those with unavailable outcome data.

Statistical analysis

Data extracted and collated from hospital records was checked and analyzed using Statistical Package for Social Sciences (SPSS) version 22.0 (IBM Corp., Armonk, NY, USA). The descriptive data were presented as mean (SD) for continuous variables and proportions (%) for categorical variables. Descriptive data were presented as contingency tables, Fisher's exact or Chi-square test was used to analyze categorical variables, and student's t-test (unpaired) for continuous variables. A p-value less than 0.05 was considered statistically significant.

## Results

During wave 1, we admitted and managed 6,333 patients, and in wave 2, we catered to 2,080 patients. The progression of both waves, in terms of admissions and mortality over time, is presented. Table 1 summarizes the details of disease severity, hospital outcomes, and mortality rates. This table reveals that the mortality rates in wave 2 were approximately ten times those of wave 1 (TMR was 20.3% in wave 2 compared to 2.4% in wave 1; CFR was 17.7% in wave 2, contrasting with 1.5% in wave 1).

**Table 1:** Summarizing the hospital outcomes and mortality rates during the two waves. LAMA: Leave against medical advice; TMR: Total hospital mortality rate; CFR: Case fatality rate.

	Wave 1	Wave 2
Total admissions	6333	2080
Total discharges	6095	1589
Excluded: Transfer out/LAMA	88	85
Total deaths	150	406
TMR	2.4 %	20.3%
COVID-19 attributed deaths	93	355
CFR	1.5 %	17.7%

**Figure 1:**
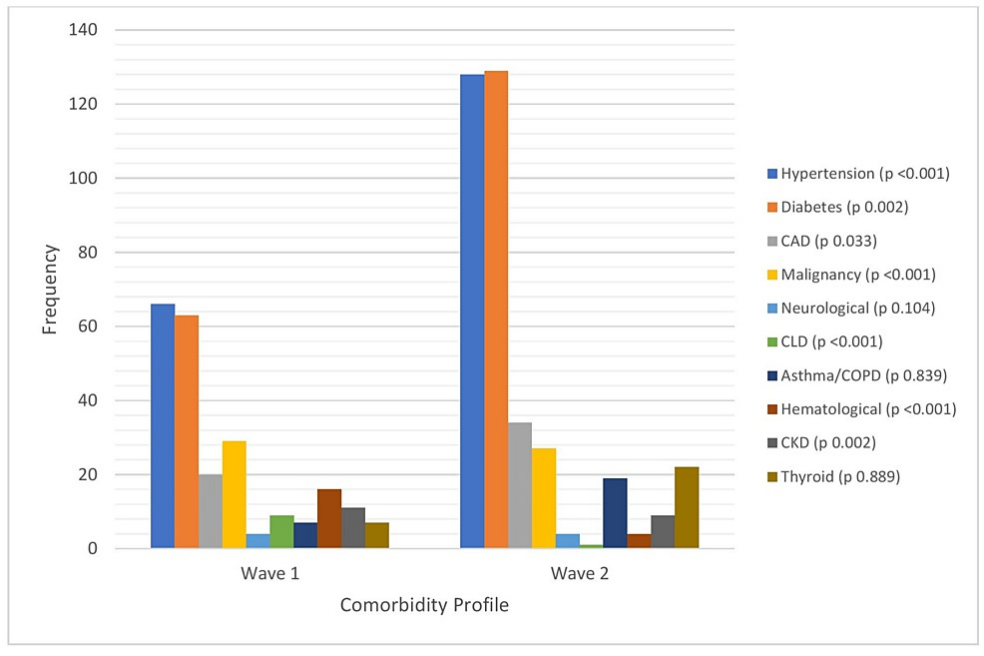
Comparison of co-morbidities among mortality cases in the two waves. CAD - Coronary Artery Disease, CLD - Chronic Liver Disease, CKD - Chronic Kidney Disease.

Table 2 tabulates the patient demographics and clinical status at the time of admission for mortality cases in both waves, while Figure 1 depicts the comorbidity profile of these patients. As evidenced by Table 2, the mean age and gender distribution of mortality cases are comparable in both waves. Furthermore, no significant difference was observed in mortality rates across similar age groups between the two waves.

**Table 2:** Summarizing the patient demographics in the two waves.

Variable	Wave 1	Wave 2	P-value
Age, Mean (SD)	58.17 (15.82)	57.57 (15.36)	0.698
Gender: Male, N (%)	100 (73.5)	274 (67.5)	0.187
Age: ≤16 years	3 (2.2)	3 (0.7)	0.304
Age: 17-39 years	17 (12.5)	59 (14.5)	
Age: 40-64 years	62 (45.2)	205 (50.5)	
Age: ≥65 years	54 (39.7)	139 (34.2)	
Comorbidity, Present	123 (90.4)	248 (61.1)	<0.001

Our results highlight subtle yet significant differences in patient profiles and mortalities between the two waves. Deaths in the first wave were more often seen in patients with chronic comorbid conditions; 90% of deaths in wave 1 had one or more comorbidities compared to 61% in wave 2 (p <0.001, Table 2). The mean duration from the onset of symptoms to death was shorter in the second wave (18 days in wave 1 vs. 16 days in wave 2, p=0.035, Table 3). However, the time taken to be admitted to our hospital after receiving a positive report was significantly shorter in the second wave (a mean of 5.7 days in wave 1 vs. 4.1 days in wave 2, p<0.001, Table 3). Compared to the first, mortality cases in the second wave were more frequently presented with severe disease upon hospital admission (78% of all deaths in wave 2 were classified as severe disease compared to 58% in wave 1, p<0.001). A significantly larger proportion of patients in the second wave presented requiring oxygen (42.8% in wave 2 versus 9.6% in wave 1, p<0.001, Table 3). Furthermore, those on oxygen during the second wave exhibited more severe hypoxia; the SpO2 at presentation for these cases was significantly lower in the second wave (83%) compared to the first (89%, p=0.05, Table 3).

**Table 3:** Comparing the duration of hospital stay, the presentation of hypoxia, and the progression of the disease to death.

Variable	Wave 1	Wave 2	P-value
Period: Hospital stay (days), Mean (SD)	10.60 (8.91)	9.05 (8.26)	0.064
Period: Symptom onset to death (days)	18.29 (8.76)	16.34 (9.36)	0.035
Period: Covid-19 test to admission (days)	5.70 (5.91)	4.10 (4.06)	0.001
Period: Covid-19 test to death (days)	13.75 (8.94)	12.70 (9.28)	0.265
SPO_2_ at Presentation: Room Air	84.70 (12.83)	82.66 (13.76)	0.557
SPO_2_ at Presentation: On Oxygen	89.75 (6.35)	83.35 (15.56)	0.050
Clinical status at presentation: Mild, N (%)	32 (25.8)	54 (13.6)	<0.001
Clinical status at presentation: Moderate	20 (16.1)	32 (8.1)	
Clinical status at presentation: Severe	72 (58.1)	310 (78.3)	
Presenting on oxygen	12 (9.6)	168 (42.8)	<0.001
Hospital stay > 2 weeks	36 (26.5)	75 (18.5)	0.045

These differences in presenting features are reflected in the causes of death. Clinically, ARDS and hypoxic respiratory failure were significantly more common as the immediate causes of death in the second wave (Table 4). Analysis of causes of death records indicated a significantly higher number of patients had deaths directly attributed to COVID in the second wave compared to the first (93 out of 135 detailed causes identified in wave 1 and 355 out of 403 detailed causes identified in wave 2, which is 68.9% vs. 88.1% in wave 1 and 2 respectively, p <0.001).

**Table 4:** Comparing the immediate causes of death in both waves. ARDS: Acute respiratory distress syndrome.

Immediate causes of death	Wave 1, N (%)	Wave 2, N (%)	P-value
ARDS/Hypoxic respiratory failure	92 (68.1)	355 (88.1)	<0.001
Sudden cardiac event	14 (10.4)	25 (6.2)	0.758
Septic shock	13 (9.6)	4 (1.0)	<0.001
Cardiogenic shock	4 (3.0)	-	-
Liver failure	4 (3.0)	1 (0.2)	0.867
Others	8 (5.9)	18 (4.5)	0.993

## Discussion

The main finding from our data is the staggering increase in mortality in wave 2 (TMR of 2.4% in wave 1 and 20.3% in wave 2). We could not find any direct comparison of in-hospital mortality between the two COVID-19 waves in the Indian scenario. This high mortality in the second wave may be attributed to cases due to the more severe delta variant of the virus. Our hospital, a tertiary care COVID-19 setup catering mainly to Delhi and North India, observed a surge in the delta variant cases during the second wave. The difference in total admissions between the two waves can be attributed to clinical and epidemiological variations, differential admissions, and more asymptomatic/mild patients admitted during the first wave for isolation purposes, which were home-quarantined during the second wave. The average population-based CFR reported globally during the initial period of the pandemic was 3-4% [9]. A meta-analysis that examined in-hospital mortality due to COVID-19 from January to August 2020, encompassing 58 studies, calculated the pooled hospital mortality rate at 18.88% from a total of 122,191 patients [12]. Thus, the data from our hospital during the second wave aligns with globally reported hospital mortality rates. The low mortality figures from India have been widely questioned in literature, and it is estimated that there is an underreporting of infections and deaths from India by a factor of 11.11 and 3.56 times in wave 1 and 26.77 and 5.77 times, respectively in wave 2 [10]. Analyzing the demographics of our hospital population and comparing it with worldwide data, the mean age of mortality cases was comparable in both waves (58.17 ± 15.82 years in wave 1 and 57.57 ± 15.36 years in wave 2, p = 0.698). This mean age more or less corresponds to the mean age of 54.16 ± 12.33 years reported by a systematic review analyzing predictors of mortality in 114 studies, including 310,494 COVID-19 patients [13]. We found no significant difference in the gender and age group distribution of deaths. The systematic review on predictors of mortality [13] also states no significant gender difference in deaths but attributes increased age as an independent risk factor for death. However, male sex and age >65 years have been reported to be independent predictors of mortality [12].

When comparing the mortality of hospitalized cases between the two waves, a significantly larger number of patients were categorized as severe at presentation during wave 2 (58.1% in wave 1 versus 78.3% in wave 2, p <0.001). The proportion of hypoxic patients at presentation was also significantly higher in wave 2, and the mean SpO2 recorded at the time of admission for patients on oxygen was notably lower in wave 2 (see Table 3). In comparing the comorbidities, wave 1 had a larger proportion of patients with comorbidities (Table 2). The most frequently seen comorbidities were hypertension and diabetes in both waves, but wave 2 had a significantly larger proportion (Figure 1). Disorders like solid organ malignancy, chronic kidney disease, and chronic liver disease were more in wave 1. These findings also indicate that severe COVID-19-related ARDS was the main cause of mortality in wave 2, although a significantly lesser proportion of patients had comorbidities. Similarly, differences in comorbidity status may have contributed to differential mortality rates between COVID-19 waves.

The progression of the disease to death was significantly faster in wave 2 (18.29 ± 8.76 days-wave 1, 16.34 ± 9.36 days-wave 2, p = 0.035). These values roughly correspond to the reported mean duration of 17.8 days from symptom onset to death in a database of patients from China [14]. Our patients' comorbidity and presenting symptom profiles match the independent risk factors predicting death reported in the literature [12,13,15].

A few countries have made attempts to compare the characteristics of two waves of COVID-19 in their populations. Contrary to our findings, countries like Japan [4] and Spain [5,16] have reported lesser severity and morbidity in the second wave. However, hospital-based mortality data from all hospitals in South Africa reported a 31% increase in mortality in the second wave [17]. We observed approximately a 17.9% increment in hospital-based mortality rates compared to South Africa, with a 31% increase probably due to different COVID-19 variants and public health responses. A study comparing raw CFRs in the two waves in 53 countries [6] reported that 43 out of 53 studied nations reported a drop in fatalities in the second wave. The reasons for these differences in the worldwide literature may be multifactorial variants of the virus affecting each nation, population densities and social dynamics affecting the spread of disease, the health care infrastructure to handle caseloads, and others. Our study is one of the first to come out with hospital-based mortality data from India. India is among the most affected nations in the world regarding COVID-19 caseload. However, no concrete data exists differentiating the two waves; population-based data from Andhra Pradesh and Tamil Nadu states a CFR ranging from 0.05% to 16.6% [18]. We have attempted to put forth concrete data on mortality from a tertiary care COVID-19 center in India and compare the disease characteristics from the two waves. However, we still lack multicentric data collection and collation to bring forth the exact characteristics of in-hospital mortality in India's two waves of COVID-19. These lacunae in our data collection and reporting should be looked into and corrected prospectively as the threat of a new surge in cases looms in the form of newer virus variants [19]. Findings from our study may be used prospectively to generate data entry indices that may be used across all COVID-19 facilities dealing with possible surges in cases in India and generate homogenous data to know the real extent of this pandemic and its effect on our healthcare infrastructure.

## Conclusions

This study is one of the first concrete attempts to compare the in-hospital mortality from the two waves of COVID in India. The mortality figures reported from India are mostly population-based estimates. Therefore, the data collected in the study provides information on the actual in-hospital mortality from a dedicated standalone COVID-19 care center. The study reflects variations in case presentations and clinical outcomes between COVID waves and provides for better planning of hospital services during epidemics. The study is an important contribution to the variable worldwide data on differential COVID-19 disease patterns. 

The study recommends the development of a customized strategy to mitigate the impact of the COVID-19 epidemic across all levels of healthcare and policymaking. There is a pressing need for a standard reporting format and uniform definitions for the grades of COVID-19 severity and criteria for hospital admission. A standardized format for reporting COVID-19-attributable mortality to a central database should be established to ensure consistent reporting and enhanced data collection, facilitating accurate interpretation and comparison.

## Appendices

Author contributions

Saurabh Vig served as the chief investigator, taking charge of planning and conducting the study, data analysis, interpretation of results, and both drafting and finalizing the manuscript. Jitendra Kumar Meena was instrumental in collating data from both waves, handling statistical analysis, tabulation, and contributing to the drafting and finalization of the manuscript. Abhishek Kumar and Puneet Rathore both worked on data collection for wave 1. Swati Bhan, Nishkarsh Gupta, and Sushma Bhatnagar were pivotal in planning the study, with Bhan and Bhatnagar also involved in analyzing results, literature search, writing discussion, and finalizing the manuscript, respectively. Prashant Sirohiya focused on result analysis and discussion drafting. Gitartha Goswami, Arunmozhimaran Elavarasi, and Hari Krishna Raju Sagiraju contributed to data collection, tabulation, and literature search for wave 2. Clinical coordination of the study was managed by Brajesh Kumar Ratre, Anuja Pandit, Ram Singh, Balbir Kumar, Rakesh Garg, Ved Prakash Meena, Saurav Sekhar Paul, Anant Mohan, Randeep Guleria, and Sushma Bhatnagar, with some of them also handling literature review, data filtration, result analysis, and manpower management.

All authors played a significant role in the final manuscript writing and satisfy the ICMJE authorship criteria. Each member was integral to the study's management and conduct.
